# The effect of dietary bacterial organic selenium on growth performance, antioxidant capacity, and Selenoproteins gene expression in broiler chickens

**DOI:** 10.1186/s12917-017-1159-4

**Published:** 2017-08-18

**Authors:** A. M. Dalia, T. C. Loh, A. Q. Sazili, M. F. Jahromi, A. A. Samsudin

**Affiliations:** 1 Department of Animal Science, Faculty of Agriculture, Universiti Putra Malaysia, 43400 Serdang, Selangor Malaysia; 2 Department of Animal Nutrition, Faculty of Animal Production, University of Khartoum, Khartoum, Sudan; 3 Institute of Tropical Agriculture, Universiti Putra Malaysia, 43400 Serdang, Selangor Malaysia

**Keywords:** Broiler, Selenium, Organic, Bacteria, Antioxidant, Selenoprotein

## Abstract

**Background:**

Selenium (Se) is an essential trace mineral in broilers, which has several important roles in biological processes. Organic forms of Se are more efficient than inorganic forms and can be produced biologically via Se microbial reduction. Hence, the possibility of using Se-enriched bacteria as feed supplement may provide an interesting source of organic Se, and benefit broiler antioxidant system and other biological processes. The objective of this study was to examine the impacts of inorganic Se and different bacterial organic Se sources on the performance, serum and tissues Se status, antioxidant capacity, and liver mRNA expression of selenoproteins in broilers.

**Results:**

Results indicated that different Se sources did not significantly (*P* ≤ 0.05) affect broiler growth performance. However, bacterial organic Se of T5 (basal diet +0.3 mg /kg feed ADS18 Se), T4 (basal diet +0.3 mg /kg feed ADS2 Se), and T3 (basal diet +0.3 mg /kg feed ADS1 Se) exhibited significantly (*P* ≤ 0.05) highest Se concentration in serum, liver, and kidney respectively. Dietary inorganic Se and bacterial organic Se were observed to significantly affect broiler serum ALT, AST, LDH activities and serum creatinine level. ADS18 supplemented Se of (*Stenotrophomonas maltophilia*) bacterial strain showed the highest GSH-Px activity with the lowest MDA content in serum, and the highest GSH-Px and catalase activity in the kidney, while bacterial Se of ADS2 (*Klebsiella pneumoniae*) resulted in a higher level of GSH-Px1 and catalase in liver. Moreover, our study showed that in comparison with sodium selenite, only ADS18 bacterial Se showed a significantly higher mRNA level in GSH-Px1, GSH-Px4, DIO1, and TXNDR1, while both ADS18 and ADS2 showed high level of mRNA of DIO2 compared to sodium selenite.

**Conclusions:**

The supplementation of bacterial organic Se in broiler chicken, improved tissue Se deposition, antioxidant status, and selenoproteins gene expression, and can be considered as an effective alternative source of Se in broiler chickens.

## Background

In living organisms, natural antioxidant system protects cells from the action of harmful free radicals [1]. The antioxidant system components include: natural fat-soluble antioxidants such as vitamin E, water-soluble antioxidants like ascorbic acid, and antioxidant enzymes: glutathione peroxidase (GSH-Px), catalase (CAT) and superoxide dismutase (SOD) [2]. Moreover, Selenium (Se) micronutrient is considered a functional part of the antioxidant system, which acts via selenoproteins. At least 25 selenoproteins have been identified in chicken, which contain a selenocystein amino acid as a unique structural part [3]. Consequently, selenocysteine has a specific role in different selenoenzymes as active site for their catalytic activity. Selenoenzymes which have been identified in animals and humans include: glutathione peroxidases, thioredoxin reductases, and iodothyronine deiodinases [4]. Glutathione peroxidase is a Se-dependent enzyme involved in the antioxidant system; it is the main enzyme which helps to control free radical formation via reduction of hydrogen peroxide and lipid peroxide to water and the corresponding alcohol [5]. Addition to that, selenoprotein W plays an antioxidant function in chicken myoblasts [6]. Four endoplasmic reticulum resident selenoprotein genes (Sepn1, Selk, Sels, and Selt) were related to oxidative damages in broiler chicken muscles [7].

Selenoproteins synthesis is affected by the nutritional level of the Se. Many studies have proven that dietary Se supplementation regulates the expression of selenoproteins in most animal tissues. It has been shown that Se deficient diet decreases the expression of 25 selenoproteins in the chicken muscular stomach [3]. In broiler kidney, the mRNA levels of 14 selenoprotein genes (Dio1, Dio2, GSH-Px3, Sepp1, SelH, SelI, SelK, Sepn1, SelO, SELW1, Sep15, SelT, SelU, and SelS) were down-regulated, and 9 selenoprotein genes (GSH-Px1, GSH-Px2, GSH-Px4,SelPb, Txnrd1, Txnrd2, Txnrd3, SPS2, and SelM) were up-regulated due to low Se diet, while Dio3 and Sepx1 mRNA levels were not affected [8]. In addition, Se supplemented diet resulted in a significant elevation in mRNA level of SELW1 in broiler liver [9]. However, broiler liver gene expression of GSH-Px4 was down-regulated as a consequence of Se-enriched diet [10]. Usually, Se is supplemented to the broiler diets in the form of inorganic Se (sodium selenite), or organic Se (natural Se sources). According to Surai [1], organic Se is more bioavailable in the animal tissues than inorganic forms, and has significant biochemical and physiological benefits. Previous studies have demonstrated that different sources of Se may result in different metabolic effects in the animal tissues [11, 12]. According to Yuan et al. [11], supplementation of organic Se in the form of Se-yeast and selenomethionine to broiler chicken showed significant up-regulation in the liver GSH-Px1 and TXNDR1 mRNA levels compared to sodium selenite. Therefore, the present study sought to determine the impact of various bacterial sources of Se as an alternative organic Se compared to the inorganic Se on growth performance, antioxidant capacity, biochemical status, and mRNA expression of some selected selenoproteins in broiler chicken.

## Methods

### Chemicals

The antioxidant assay kits were purchased from bioassay system (USA). The inorganic sodium selenite Na_2_SeO_3_, ≥99%, was sourced from Sigma-Aldrich Chemical Co., St. Louis, MO, USA. Reagents used for real time PCR were purchased from Qiagen Biotechnology Malaysia Sdn. Bhd and Thermo Scientific Fermentas. All reagents for the Se assay were of analytical grade.

### Bacterial strains

The bacterial strains used in this study were isolated as Se enriched bacteria from rumen fluid (ADS1 and ADS2) and hot spring water (ADS18) (Selangor, Malaysia), and identified as *Enterobacter cloacae* (ADS1), *Klebsiella pneumoniae* (ADS2), and *Stenotrophomonas maltophilia* (ADS18). All of them had high ability in accumulating organic Se in their cells when grown in Se-enriched medium according to our previous finding.

### Preparation of bacterial organic se

The 30% glycerol stock culture of ADS1, ADS2, and ADS18 strains was used to prepare aliquots fresh culture (24 h) after three times reviving at the Laboratory of Microbiology, Department of Animal Science at the Faculty of Agriculture, Universiti Putra Malaysia (UPM). The commercially available media, nutrient broth supplemented with 10 μg/mL sodium selenite were used for all strains inoculation. Then, ADS1 and ADS2 strains were incubated at 39 °C and ADS18 at room temperature for 24 h. This was followed by single colony sub-culturing using spread plate technique and incubation for 24 h at the same stated temperature. A single colony was then inoculated into 10 mL inorganic Se- enriched nutrient broth and subjected to incubation for 24 h. It was then sub-cultured two times, after which it was ready for use as an inoculum. An inoculum containing 1 × 10^6^ of isolated bacterial cells was inoculated into the same media and followed by incubation for 24 h at static temperature. The next step was centrifuging the culture at 6000 rpm for 15 min to harvest the bacterial pellets enriched with Se which were then washed two times using deionized water to remove any inorganic Se in the bacterial cells [13]. Selenium-enriched bacterial cells were collected and lyophilized at −20 °C. Furthermore, the collected bacterial biomass was subjected to ultra-sonication to disrupt the bacterial cell walls and release their organic Se-content. Sonication was performed using ice water bath for 90 cycles, with 5 s on and 5 s off. Then the sonicated Se- enriched biomass was lyophilized and kept at −20 °C prior to use it as Se source in the feeding trial.

### Birds and experimental procedure

A total of 180 one-day-old female (Cobb 500) broiler chicks averaging 40 ± 0.13 g in body weight were sourced from a commercial hatchery and randomly allocated to five treatments, each of which was replicated six times with 6 birds per replicate. The treatment groups included T1 = basal diet (negative control), T2 = basal diet +0.3 mg/Kg feed inorganic Se Na_2_SeO_3_ (positive control), T3 = basal diet +0.3 mg /kg feed ADS1 Se, T4 = basal diet +0.3 mg /kg feed ADS2 Se, T5 = basal diet +0.3 mg /kg feed ADS18 Se. Starter and finisher basal diets (Table 1) were prepared in line with the nutritional requirements of broilers and according to NRC (National Research Council) (1994) standards, except for Se which were supplemented as 0.3 mg/kg feed according to Surai, [1]. Starter diet was offered from 0 to 3 weeks old and finisher from 4 to 6 weeks old. Water and feed were given ad libitum to all the chickens until 42 days of age. The study was conducted in compliance with the research policy guidelines of UPM on Animal Welfare and Ethics.

**Table 1:** Ingredients and nutrient content of the basal diet

Ingredients	Starter	Finisher
	%	%
Corn	52.5	56.250
Palm oil	5.00	6.00
Soybean meal (44% cp)	32.50	30.00
Fish meal (58% cp)	5.15	3.25
L-Lysine	0.25	0.25
DL-Methionine	0.25	0.25
Dicalcium phosphate 18% ^a^	1.60	1.85
Calcium carbonate	0.60	0.35
Salt	0.30	0.30
Mineral Premix^b^	0.15	0.15
Vitamin Premix^c^	0.10	0.10
Toxin Binder^d^	0.15	0.15
Choline Chloride	0.10	0.10
Wheat pollard (QL)	0.135	1.00
Calculated nutrient content (g/kg DM) ^e^		
ME (MJ/Kg)	12.9	13.20
Crude protein	22.04	20.09
Crude fat	7.57	8.004
Calcium	1.189	1.0440
Phosphorus	0.786	0.768
Avail. P for Poultry	0.472	0.450
Analyzed Se (mg/kg)^f^	<0.09	<0.09

^a^ di calcium phosphate provides phosphorus and calcium in a ratio of 1:1

^b^Mineral premix provided the following per kg diet: iron 120 mg, manganese 150 mg, copper 15 mg, zinc 120 mg, iodine 1.5 mg, and cobalt 0.4 mg

^c^Vitamin premix provided the following per kg diet: Vitamin A (retinyl acetate) 10.32 mg, cholecalciferol 0.250 mg, vitamin E (DL-tocopheryl acetate) 90 mg, vitamin K 6 mg, cobalamin 0.07 mg, thiamine 7 mg, riboflavin 22 mg, folic acid 3 mg, biotin 0.04 mg, pantothenic acid 35 mg, niacin 120 mg and pyridoxine 12 mg

^d^Toxin binder contains natural hydrated sodium calcium aluminium silicates

^f^The Se content measured using ICP.MS

^e^The diets were formulated using feedlive International software (Thailand)

### Growth performance

The body weight (BW) and pen feed intake (FI) of individual birds were recorded weekly, and weight gain (WG) and feed conversion ratio (FCR) were calculated. FCR was calculated as follows: FCR = total feed consumed by birds/total weight gain.

### Slaughtering, blood and tissues sampling

At day 42 of feeding trial, 12 birds per treatment were taken as representative samples and were slaughtered to collect blood and tissue samples. Blood samples were collected directly into plain serum bottles, kept at room temperature for 10 min, and then centrifuged at 4000 rpm for 10 min; resultant supernatant was collected and stored at −80 °C until further analysis. Liver and kidney tissue samples were obtained and frozen directly in liquid nitrogen and stored at −80 °C to await analysis. Around 100 mg of liver tissues was collected immediately in RNA-later Stabilization Reagent (Qiagen, Germany) and processed for storage at −80 °C, following the manufacturer’s instructions.

### Determination of biochemical parameters

Biochemical parameters, such as serum total protein (TP), albumin (AL), aspartate aminotransferase (AST), alanine aminotransferase (ALT), lactate dehydrogenase (LDH) blood urea nitrogen (BUN), and serum creatinine (Cr), were established with the auto-blood biochemical analyzer (Automatic Analyzer 902, Hitachi, Germany) using the appropriate kit. Serum globulin (G) and albumin/ globulin ratio (A/G) were calculated as follows: G = total protein - albumin, A/G = albumin/globulin. All samples were tested in duplicate.

### Assay of se content in tissues

For Se analysis in serum and tissue samples, 0.5 mL of serum and 0.5 g tissue samples were digested by mixing with 5 mL HNO_3_ and 1 mL H_2_O_2_ in a digestion tube using microwave digestion system. To the remaining 1 mL of the digests, deionized water was added to produce a 10 mL solution. The Se concentration was measured immediately in the solution using the inductively coupled plasma mass spectrometer (Agilent Technologies, Santa Clara) following the method reported by Wahlen et al. [14].

### Determination of antioxidant enzymes activity

Total antioxidant (T-AOC), glutathione peroxidase activity (GSH-Px), superoxide dismutase activity (SOD), catalase activity, and the concentration of malondialdehyde (MDA) were measured in the serum and liver, and kidney tissues. Liver and kidney tissues were homogenized on ice using TBS buffer and centrifuging at 3000×g for 10 min at 4 °C, and the resultant supernatant was collected for enzymes measurement. Antioxidant enzymes analysis was performed using BioAssay Systems Commercial kit.

### Determination of selenoprotein mRNA expression

For selenoproteins mRNA determination, 30 mg of RNAlater preserved liver samples were used for total RNA isolation with the high pure RNA Tissue Kit (RNeasy mini kit, Qiagen, USA). The samples were homogenized appropriately in the lysis buffer (Qiagene) following the manufacturer’s instructions. Qualitative and quantitative assessments of the isolated RNA were carried out on ND-1000 NanoDrop (NanoDrop Technologies, USA) spectrophotometer. Only samples with more than 100 ng RNA and absorbance ratios of A260/280 and A260/230 of around (> 1.8) were selected for further manipulation. The RNA was reverse transcribed into cDNA with the transcription first strand cDNA Synthesis Kit (one-step RT-PCR kit, Qiagen, USA) following the procedure recommended by the manufacturer.

Primers for gene expression were designed (First Base, Malaysia) based on published *Gallus gallus* sequences (Table 2). The relative mRNA abundances of 6 genes were assayed (glutathione peroxidase 1, GSH-Px1; glutathione peroxidase 4, GSH-Px4; deiodinase1, DIO1; deiodinase 2, DIO2; selenoprotein W1, SELW1; thioredoxin reductase 1, TXNRD1). The reaction was done in a Bio-Rad thermal cycler (MyCycler, Germany). The RT-PCR conditions included: (1) reverse transcription, 30 min, 50 °C; (2) initial PCR activation step, 15 min, 95 °C; (3) 3-step cycling for 40 cycles, each cycle consisting of denaturation for 30 s at 94 °C followed by annealing for 30 s at 52–57 °C and extension for 1 min at 72 °C. The linearity of response was ensured and the saturation of the reaction was prevented through optimization of the template concentration and the cycle number. To standardize the expression data, the GADPH mRNA fragment was employed as internal standard (housekeeping gene). The results were standardized to the levels achieved for the β-actin gene. It was carried out by taking the ratio of the obtained value for the gene of interest to that of GADPH and then related to the control. 2-ΔΔCt (ΔΔCt = ΔCt Test sample-ΔCt Calibrator sample) to calculate the relative mRNA expression.

**Table 2:** Genes and primers used for relative quantification by real time PCR (qPCR) in the liver of chicken

Gene^a^	Primer sequence (5′ − 3)^b^	Fragment bp
GAPDH	Forward: 5′-AATGAGAGGTTCAGGTGCCC-3′	150
	Reverse: 5′-ACCAGACAGCACTGTGTTGG-3′	
GSH-Px1	Forward: 5′-GCGACTTCCTGCAGCTCAACGA-3′	99
	Reverse: 5′-CGTTCTCCTGGTGCCCGAAT-3′	
GSH-Px4	Forward: 5′-CGGTGAATTACACTCAGCTCGT-3′	123
	Reverse: 5′-CTTTGATCTGCGCGTCGTCC-3′	
DIO1	Forward: 5′-AAGCTGCACCTGACCTTCATT-3′	138
	Reverse: 5′-TTGTTTCTGAAGGCCCATCCA-3′	
DIO2	Forward: 5′-CAGTGTAATCCACATAGCCA-3′	137
	Reverse: 5′-CTGAGCCAAAATTAACCACC-3′	
SELW1	Forward: 5′-CTCCGCGTCACCGTGCTCT-3′	155
	Reverse: 5′-CTGCCCACCGTCACCTCGAAC-3′	
TXNDR1	Forward: 5′-ACTGGATGACTATGACCGAA-3′	103
	Reverse: 5′-TATGCATTCTCATACGTGAC-3′	

^a^ Abbreviation: GAPDH, glyceraldehyde-3-phosphate dehydrogenase; GSH-Px1,Glutathione peroxidase1; GSH-Px4, Glutathione peroxidase4; DIO1, iodothyronine deiodinase1; DIO2, iodothyronine deiodinase2; SELW1, selenoproteins w; TXNDR1, thioredoxin reductase

^b^primers used for qPCR designed based on pupplished sequences [54]

### Statistical analysis

An ANOVA was conducted using six replicates per means. Differences between treatments were scrutinized with one-way ANOVA (SAS Institute, 1996). Duncan test was used to determine the significant differences among the treatment groups at a significant level (*P* < 0.05). The data of serum and tissues Se concentration and antioxidant enzymes were analyzed employing GLM procedure applicable for Completely Randomized Design (SAS, 1996). Treatment differences were established by orthogonal contrasts

1. Basal diet vs. Se supplemented diets,
2. Sodium selenite vs. bacterial organic Se,

Values of *P* < 0.05 were considered significant.

## Results

### Growth performance of broiler chicken

The growth performance of birds fed diverse sources of Se is presented in Table 3. No significant differences (*P* > 0.05) among the dietary treatments were observed during the experimental period. The results demonstrated that supplementation of inorganic and bacterial organic Se did not affect body weight, weight gain, feed intake, and FCR ratio.

**Table 3:** Growth performance (means ± SE) at week 6 of treatments supplemented with different sources of bacterial organic Se

	Dietary Treatments^a^						
Parameters^b^	T1	T2	T3	T4	T5	SEM	*P*
BW (g)	2007.9	2082.1	2054.8	2075.4	2093.9	21.65	NS
DWG (g)	1965.3	2039.5	2012.2	2064.2	2081.3	21.6	NS
FI (g)	3169.1	2983.6	2969.2	3011.1	3248.0	69.3	NS
FCR	1.61	1.46	1.47	1.46	1.56	0.04	NS

*NS* No significant differences

^a^T1; basal diet, T2; basal diet +0.3 mg/ kg feed sodium selenite, T3; basal diet +0.3 mg/ kg feed ADS1 Se, T4; basal diet +0.3 mg/ kg feed ADS2 Se, T5; basal diet +0.3 mg/ kg feed ADS18 Se

^b^BW; body weight, DWG; daily weight gain, FI; feed intake, FCR; feed conversion ratio

### Serum and tissues se concentration

Table 4 shows the Se content in the serum, liver, and kidney of broiler chicken supplemented with inorganic Se and different sources of bacterial organic Se for 42 days. Selenium supplemented diets versus basal diet showed significant increase and deposition of Se in serum and tissue samples compared to negative control (T1). Moreover, bacterial organic Se in broiler feed resulted in a significant (*P* < 0.05) Se deposition in the liver and kidney tissues compared to inorganic Se (T2). Serum Se concentrations were significantly (*P* < 0.0001) higher in T2, T4, and T5 than T1, but, T5 showed the highest level among the dietary treatments. In liver and kidney tissues, the highest Se levels were observed in T4 and T3 bacterial organic Se for liver and kidney respectively. Additionally, Se de osition in the liver showed no difference between birds receiving dietary supplementation of inorganic Se and bacterial organic Se of T3 and T5 compared to negative control, but in kidney all Se supplemented diets showed significant (*P* < 0.0001) difference compared to birds supplemented with basal diet.

**Table 4:** Selenium concentration in serum and tissues of broiler chicken fed different Se sources

Parameters	Dietary treatments^a^					SEM	*P* value		
	T1	T2	T3	T4	T5		Anova	B	O
Serum μg/l	38.79^c^	50.94^b^	42.07^c^	58.61^ab^	61.21^a^	2.14	<.0001	0.0002	0.3705
Liver μg/kg	124.9^b^	146.3^ab^	153.5^ab^	170.4^a^	133.5^b^	5.03	0.0246	0.0226	0.0476
Kidney μg/kg	114.9^c^	136.5^b^	178.5^a^	148.4^b^	143.2^b^	4.98	<.0001	<.0001	0.0155

*B* Basal diet VS Se supplemented diets, *O* Organic Se VS inorganic Se, *p* < 0.05 = significant differences

^a-c^ Means with different letter within a row differed significantly

^a^T1; basal diet, T2; basal diet +0.3 mg/ kg feed sodium selenite, T3; basal diet +0.3 mg/ kg feed ADS1 Se, T4; basal diet +0.3 mg/ kg feed ADS2 Se, T5; basal diet +0.3 mg/ kg feed ADS18 Se

### Serum biochemical profile in broiler chicken fed different sources of se

The serum biochemical parameters of broiler fed different sources of Se are shown in Table 5. Selenium supplementation as inorganic or bacterial organic forms did not affect serum total protein, albumin, globulin, albumin/ globulin ratio, and urea. However, the differences were significant (*P* < 0.05) among the treatments in serum AST, ALT, LDH and Creatinine levels. The activities of AST, ALT, LDH enzymes and creatinine level in serum were decreased in birds fed Se compared to basal diet, ALT level was decreased significantly (*P* < 0.05) in T4 and T5 compared to T1 and T2, while T3 showed insignificant effect among all treatments. AST level was the highest in T1 with no significant difference compared to inorganic Se (T2) and bacterial organic Se (T4 and T5), but the level was significantly higher than T3. In addition, T3 showed the lowest (*P* < 0.05) level of LDH compared to all treatments. Moreover, dietary Se as inorganic and bacterial organic form reduce the serum creatinine level compared to negative control group T1, and the difference was significant (*P* < 0.05) compared to T2, T3, and T4 while no significant differences were observed compared to T5.

**Table 5:** Effects of dietary supplementation of inorganic and bacterial organic Se on serum biochemical profiles in broiler

Parameters	Dietary Treatments^a^						
	T1	T2	T3	T4	T5	SEM	*P*
T. Protein (g/L)	29.0 ^a^	27.8 ^a^	21.6 ^a^	21.3 ^a^	28.3 ^a^	1.29	NS
Albumin (g/L)	19.5 ^a^	17.6 ^a^	16.7 ^a^	17.0 ^a^	21.4 ^a^	0.66	NS
Globulin (g/l)	9.5 ^a^	10.2 ^a^	4.9 ^a^	4.3 ^a^	6.9 ^a^	0.87	NS
Albumin/Globulin ratio	2.5	2.1	4.6	3.8	3.70	0.98	NS
ALT (U/L)	7.02 ^a^	5.28 ^ab^	5.66 ^ab^	4.00 ^b^	5.12 ^b^	0.35	*
AST (U/L)	293.2 ^a^	264.3 ^ab^	203.9 ^b^	234.0 ^ab^	267.1 ^ab^	10.8	*
LDH (U/L)	1848.3 ^a^	1755.8 ^a^	1456.3 ^b^	1749.5 ^a^	1927.6 ^a^	47.0	*
Creatinine (umol/L)	28.3 ^a^	21.5 ^bc^	17.8 ^c^	21.4 ^bc^	25.0 ^ab^	1.1	*
Urea (umol/L)	0.64 ^a^	0.53 ^a^	0.53 ^a^	0.58 ^a^	0.67 ^a^	0.02	NS

^a^T1; basal diet, T2; basal diet +0.3 mg/ kg feed sodium selenite, T3; basal diet +0.3 mg/ kg feed ADS1 Se, T4; basal diet +0.3 mg/ kg feed ADS2 Se, T5; basal diet +0.3 mg/ kg feed ADS18 Se

^a,b,c^ Means in the same row with different superscripts are significantly different

*: significant differences (*P* < 0.05). NS: No significant differences

### Antioxidant status of serum, liver, and kidney of broiler chicken

As shown in Table 6, the effect of bacterial organic Se supplementation on the antioxidant variables of serum, liver and kidney of broiler chicken were varied according to Se source and type of tissue. Se supplementation in contrast to basal diet induced a notable elevation (*P* < 0.05) in serum, liver, and kidney GSH-Px and catalas activity, with substantial reduction in MDA concentration, while the supplementation of bacterial organic Se in contrast to inorganic source had no obvious effect on antioxidant parameters, except in the kidney tissue where there was a significant (*P* < 0.05) increase in GSH-Px, SOD, and catalase activity with significant decrease in MDA level. Also serum SOD showed significant difference compared to inorganic Se.

**Table 6:** Effect of dietary supplementation of bacterial organic Se on serum, liver, and kidney antioxidant status of broilers

Parameters	Dietary treatments ^1^					SEM	*P* value		
	T1	T2	T3	T4	T5		Anova	B	O
Serum									
T-AOC^2^	647.35	675.78	705.45	655.18	653.95	12.90	0.654	0.465	0.904
GSH-Px^3^	8.50^b^	11.78^a^	11.46^ab^	11.28^ab^	13.61^a^	0.536	0.029	0.005	0.764
SOD^4^	0.159^b^	0.166^a^	0.160^b^	0.160^b^	0.161^b^	0.001	0.027	0.112	0.004
Catalase^5^	12.064	12.642	12.588	12.325	12.363	0.081	0.149	0.039	0.271
MDA^6^	1.158^a^	0.929^b^	0.959^b^	0.869^b^	0.736^c^	0.035	<.0001	<.0001	0.1180
Liver									
T-AOC	1250.8	1306.6	1576.1	1342.7	1484.9	41.35	0.053	0.059	0.092
GSH-Px,	99.37^b^	111.23^b^	107.68^b^	154.60^a^	103.45^b^	6.117	0.009	0.039	0.376
SOD	0.1672	0.1670	0.1673	0.1673	0.1672	0.0001	0.781	0.927	0.2512
Catalase	12.279^c^	12.770^b^	12.831^ab^	13.037^a^	12.857^ab^	0.066	<.0001	<.0001	0.139
MDA	0.989^a^	0.640^b^	0.773^ab^	0.733^b^	0.800^ab^	0.039	0.044	0.007	0.141
Kidney									
T-AOC	1120.10^c^	1222.63^ab^	1263.41^a^	1122.10^c^	1182.73^cb^	16.05	0.003	0.012	0.253
GSH-Px	41.12^c^	51.26^b^	55.32^b^	47.84^bc^	72.62^a^	2.679	<.0001	0.001	0.042
SOD	0.1664^cb^	0.1663^c^	0.1665^cb^	0.1671^a^	0.1670^ab^	0.0001	0.027	0.192	0.021
Catalase	13.159^b^	13.160^b^	13.259^a^	13.217^ab^	13.284^a^	0.016	0.013	0.031	0.008
MDA	7.202^a^	7.171^a^	5.358^b^	5.124^b^	5.757^b^	0.229	<.0001	0.0003	<.0001

B = basal diet versus Se supplemented diets, O = organic Se versus inorganic Se diet,

^a,b,c^ means having different superscript along the same row for each factor are significantly different (*P* < 0.05)

^1^T1; basal diet, T2; basal diet +0.3 mg/ kg feed sodium selenite, T3; basal diet +0.3 mg/ kg feed ADS1 Se, T4; basal diet +0.3 mg/ kg feed ADS2 Se, T5; basal diet +0.3 mg/ kg feed ADS18 Se

^2^T–AOC expressed as μM Trolox Equivalents

^3^Glutathione peroxidase activity is expressed as U/L (one unit is the amount of GSH-Px that produces 1 μmole of GS-SG per min)

^4^SOD: One unit corresponds to the amount of enzyme needed to scavenges dismutation of the superoxide radical

^5^Catalase activity is expressed as U/L (one unit is the amount of catalase that decomposes 1μmole of H_2_O_2_ per min)

^6^MDA is expressesd as μM MDA equivalents

In this study, the T-AOC activity showed insignificant differences among the treatment groups in serum and liver, but the difference was significant in the kidney tissue with the highest activity in T3 and T2 compared to other groups. The GSH-Px activity was highest in all Se supplemented groups compared to the negative control in serum and examined tissues. In serum and kidney, the highest activity was observed in T5 group (13.61 and 72.62 U/L) respectively, while T4 showed the highest activity in liver (154.60 U/L). Regarding SOD activity, the level was not affected by bacterial organic Se supplementation except in kidney tissue where both T5 and T4 showed a significantly (*P* < 0.05) higher activity compared to the other groups. Moreover, bacterial organic Se showed significant elevation in catalase activity in liver and kidney, while the highest activity in liver appeared in T4 with significant difference compared to T1 and T2, while T5 and T3 showed the highest activity in kidney with significant differences compared to T1 and T2.

The serum and tissues content of MDA was significantly (*P* < 0.05) decreased by Se supplementation. Bacterial organic Se of T5 showed the significantly lowest level in serum, as well as a significantly lower level in T3, T4, and T5 compared to T1 and T2 in kidney. However, in the liver, MDA content in T2 and T4 was significantly lower than T1 group.

### Effects of bacterial organic se supplementation on the mRNA level of hepatic selenoproteins in broiler chicken

**Fig. 1:**
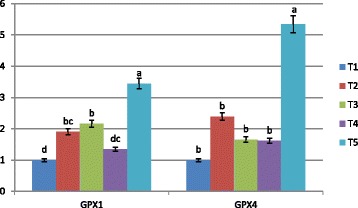
GSH-Px1 and GSH-Px4 mRNA expression in the liver of broiler chicken. Treatments: T1; basal diet, T2 basal diet +0.3 mg/kg sodium selenite, T3: basal diet +0.3 mg/kg Se of ADS1, T4; basal diet +0.3 mg/kg Se of ADS2, T5: basal diet +0.3 mg/kg Se of ADS18. Bars with no common letter differ significantly (*P* < 0.05)

**Fig. 2:**
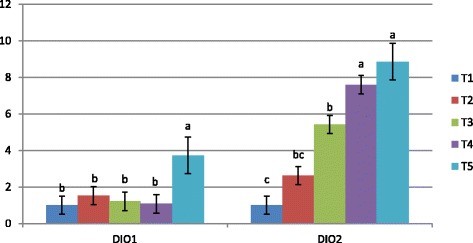
DIO1 and DIO2 mRNA expression in the liver of broiler chicken. Treatments: T1; basal diet, T2 basal diet +0.3 mg/kg sodium selenite, T3: basal diet +0.3 mg/kg Se of ADS1, T4; basal diet +0.3 mg/kg Se of ADS2, T5: basal diet +0.3 mg/kg Se of ADS18. Bars with no common letter differ significantly (*P* < 0.05)

**Fig. 3:**
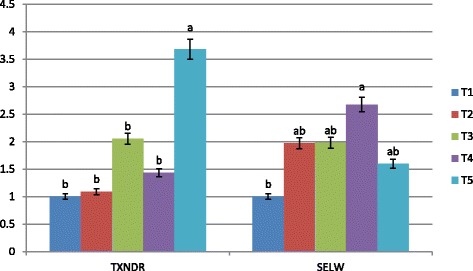
TXNDR1 and SELW1 mRNA expression in the liver of broiler chicken. Treatments: T1; basal diet, T2 basal diet +0.3 mg/kg sodium selenite, T3: basal diet +0.3 mg/kg Se of ADS1, T4; basal diet +0.3 mg/kg Se of ADS2, T5: basal diet +0.3 mg/kg Se of ADS3. Bars with no common letter differ significantly (*P* < 0.05)

To examine the effect of bacterial organic Se sources and inorganic Se form on mRNA expression of some selenoproteins, the hepatic expressions of GSH-Px1, GSH-Px4, DIO1, DIO2, TXNDR1, and SELW1 genes were investigated. Our results revealed that the expression levels of GSH-Px1 and GSH-Px4 (Fig. 1) were affected by Se supplementation. Bacterial organic Se of ADS18 (T5) showed a superior level of both GSH-Px1 and GSH-Px4 mRNA expression with significant difference (*P* < 0.05) in comparison with all other treatments. However, inorganic Se and bacterial Se of ADS1 (T3) also increased GSH-Px1 mRNA level significantly (*P* < 0.05) compared to negative control. The expression levels of DIO1 and DIO2 mRNA level in liver tissue are shown in Fig. 2. The highest expression of both genes was observed in T5 with significant difference compared to other treatment groups. However, a significant increase in DIO2 mRNA level was observed in liver of chickens fed all sources of bacterial organic Se compared to chickens fed inorganic Se and basal diet. Fig. 3 shows the mRNA expression of TXNDR1 and SELW1 genes in experimented livers. The greatest increase in TXNDR1 mRNA level was observed in the liver of T5 treatment group which was significantly (*P* < 0.01) higher than the other groups. Moreover, a very significant stability of the hepatic SELW1 mRNA level was observed in all Se supplemented groups, with no significant effect compared to basal diet group. However, the treatment group of T4 which was supplemented with bacterial Se of ADS2) showed substantial difference (*P* < 0.05) compared to negative control.

## Discussion

In the present study, supplementation of inorganic and bacterial organic Se to broiler chicken did not affect the birds’ growth performance. This finding is in agreement with the results of Perić et al. [15], Wang et al. [16], Oliveira et al. [17], and Göçmen et al. [18] who reported that different sources and levels of Se in the diet had no influence on bird BW, WG, FI, and FCR ratio, However, other studies indicated positive effects on growth performance after Se supplementation. Jiang et al. [19] reported that selenomethionine supplementation at the 0.225 mg/ kg increased the broiler’s final body weight and weight gain significantly compared to birds receiving basal diet. Zhou and Wang, [20] revealed that the supplementation of nano elemental Se in chicken for 90 days improved final BW, daily WG and FCR. Yang et al. [21] found that organic Se increased daily weight gain and feed intake in broiler chicken after 42 days of feeding. The differences in previous results and our result may be due to the fact that their basal diet was deficient in Se, whereas, in the present study, chickens received the basic requirement of Se in the basal diet. The National Research Council, (1994) maintains that the minimum dietary Se level for optimum growth and performance in broiler is 0.1 mg/kg. The Se concentration in our basal diet was around 0.09 mg/kg and this may be the reason there was no Se deficiency symptoms in our negative control.

Dietary supplementation of different sources of Se in broiler chicken affected serum, liver, and kidney Se levels. Bacterial organic Se of T5, T4, and T3 showed significantly highest Se concentration in serum, liver, and kidney respectively, compared to the basal diet and inorganic Se supplemented diet. This may be due to the bacterial Se containing proteins such as selenomethionine and selenoscystein which have the ability to be concentrated in some tissues including liver, kidney, and meat, while, inorganic Se is absorbed less efficiently and excreted in the urine at a higher level than organic Se because of their different metabolic pathways [22]. Previous studies also reported that, dietary Se resulted in an elevation of the Se concentration in the broiler liver, kidney, and breast muscle, but organic Se in the form of Se yeast showed more deposition than sodium selenite [17, 23–25]. Differences in serum and tissues Se levels between the experimented bacterial strains may attributed to the fact that all the strains in this study are able to accumulate Se- containing proteins associated with other Se- fractions such as nanoparticle elemental Se produced by *Stenotrophomonas maltophilia* and *Klebsiella pneumonia* [26, 27], and exopolysaccaride that can be produced by *Enterobacter cloacae* [28]. The abundant organic Se-molecules are Se containing proteins such as selenomethionine which is a well absorbed form and can be incorporated into body proteins in place of methionine [29]. The balance between the Se-containg proteins and other Se- fractions in the bacterial supplement may affect tissues Se deposition.

Selenium supplementation as inorganic or bacterial organic forms did not affect serum total protein, albumin, globulin, albumin/ globulin ratio, and urea. Similarly, the supplementation of Se either as Se nanoparticles or sodium selenite in the rat basal diet had no effect on serum total protein and albumin while globulin level showed improvement after Se supplementation [30]. In the same way, Yang et al. [21] reported that broiler chicks supplemented with 0.3 ppm organic Se for 42 days didn’t affect serum globulin level compared to control group. Contrary to our finding Mohapatra et al. [31] stated that supplementation of 0.3 ppm nano Se in layer chicks up to 8 weeks significantly increased total protein and serum globulin levels and also significantly lowered A:G ratio compared to control. Moreover, in the present study, dietary inorganic Se and bacterial organic Se significantly affected broiler serum ALT, AST, LDH activity and serum creatinine level. This is supported by the finding of Perić et al. [15] who found substantial reduction in both ALT and AST enzymes activity in chicken fed organic Se. The same results were obtained by Biswas et al., [32], who found a decrease in ALT and AST activities in chicks supplemented with 0.5 mg and 1 mg/kg of Se in their diet. However, Okunlola et al. [33] and Gružauskas et al. [34] indicated that serum ALT and AST increased with no differences in total protein, Albumin, Urea and creatinine in poultry supplemented with 0.5 mg of sodium selenite and others supplemented with 0.15 mg of inorganic Se and 0.35 mg of organic Se. The blood enzymes ALT, AST, LDH are used as indicators of liver and kidney oxidative damage, the serum reduction of the enzymes and creatinine levels means increasing protection against oxidative damage through an improved redox status.

Animal antioxidant system is greatly influenced by animal nutrition, and dietary Se supplementation is necessary to up-regulate the body’s glutathione pool and its Se-containing antioxidant enzymes [19]. Retention of organic Se in poultry given organic Se is better than in those given sodium selenite. Accordingly, dietary organic Se can improve antioxidant system and increase GSH-Px activity in all tissues of broiler chicken [35]. However, Glutathione peroxidase and superoxide dismutase are the main enzymatic antioxidants against toxic oxygen reduction metabolites [36]. The findings of increasing serum and tissues antioxidant enzymes activity by bacterial organic Se in this experiment were consistent with earlier results of Teo et al. [37], who revealed that the dietary supplementation of Se-enriched bacteria showed higher level of GSH-Px in heart, liver, and kidney of rats. Additionally, Se-enriched yeast as organic source enhanced antioxidative status of broilers by increasing antioxidant enzyme levels compared to sodium selenite [19]. Besides that, a study by Chen et al. [38] showed that organic Se supplementation in broiler chicken increased the activity of serum GSH-Px, SOD and total antioxidant capacity (T-AOC) more significantly than dietary sodium selenite. According to Boostani et al. [39] Se supplementation raised glutathione peroxidase (GSH-Px) activity and lowered malondialdehyde (MDA) in comparison with the control group. On the contrary, Payne and Southern [40] reported that glutathion peroxidase activity was unaffected by organic, inorganic and concentrations of Se. Some serum and tissues antioxidant enzymes in this study showed no differences between bacterial organic Se and sodium selenite, which could refer to the fact that bacterial organic Se used in this study was extracted as Se-containing proteins which mainly comprise seleno-amino acids (selenomethionine and selenocystein), therefore part of selenomethionine can be merged directly with body proteins to replace methionine instead of entering selenoproteins synthesis, while, sodium selenite can be converted to Se-Cys directly before it can be incorporated into selenoproteins enzymes [41]. Moreover, the fluctuation observed between the bacterial organic sources may be attributed to the variation in the amount of Se-Cys to Se-Met accumulated by each bacterial strain. Supplementation of bacterial organic Se also caused a significant decrease in serum and kidney MDA content compared to dietary sodium selenite, although the difference was insignificant in kidney tissues. Malondialdehyde, considered a marker of oxidative stress, is one of the final products of cell polyunsaturated fatty acid peroxidation [42]. Therefore, the decreasing of MDA by bacterial organic Se is due to the presence of Se-Met and Se-Cys which are more bioavailable than sodium selenite and can raise the levels of antioxidants and decrease the production of lipid peroxidation products.

The present study investigated the expression of selenoproteins (GSH-Px1, GSH-Px4, DIO1, DIO2, TXNDR1, and SELW1) when bacterial organic Se from three bacterial strains was supplemented to the broiler chicken and compared with inorganic source. Numerous studies have shown that expression of these genes is regulated by dietary Se intake and down-regulated in Se deficiency [43]. In Se deficiency, lower selenoprotein’s transcript levels in chicken erythrocytes was observed, while, GSH-Px, TXNDR1, selenoprotein P1 (SELP), and selenoprotein synthetase (SPS2) were highly expressed [44]. As well as, in chickens thyroid gland, DIO1, DIO2, and DIO3 selenoproteins were down-regulated [45], and GSH-Px mRNA level fell to 35–39% of Se-adequate levels in birds [46]. However, the effect of different Se sources on the expression of these genes was not fully investigated, and to our best knowledge, no study has examined the effect of bacterial Se as an organic source. In this study, the supplementation of inorganic Se has no effect on mRNA expression of all examined genes except GSH-Px1 gene. GSH-Px1 mRNA levels in sodium selenite supplemented group showed a significant difference compared with un-supplemented group. Previous studies reported that GSH-Px1 and SELW1 mRNA levels increased in responding to Se intake in poultry [47], sheep [48], and pig [49]. Dietary inorganic Se had no effect on GSH-Px4 in rat liver [43], However, Se supplementation in the form of sodium selenite resulted in increased GSH-Px1, SELW1, and TXNRD1 mRNA levels in the liver of lamb, while no change was observed in GSH-Px4 [50]. Therefore, dietary Se can regulate the expression of selenoproteins, which can eliminate reactive oxygen species through their antioxidant properties. GSH-Px is the most abundant selenoprotein in the liver, including GSH-Pxl, GSH-Px2, GSH-Px3, and GSH-Px4, and most of them are involved in the catabolism of peroxides. Our results indicate that GSH-Px1 mRNA could have higher sensitivity to regulation by Se status than GSH-Px4, and different response of mRNA expression to dietary Se might exist between selenoproteins GSH-Px1, and GSH-Px4. Moreover, our study showed that supplementation of bacterial organic Se up-regulated the mRNA expression of most examined genes significantly (*P* ≤ 0.05) compared to negative control. However, in comparison with sodium selenite, just ADS18 bacterial Se showed significantly higher mRNA level in GSH-Px1, GSH-Px4, DIO1, and TXNDR1, while both ADS18 and ADS2 showed high level of mRNA of DIO2 compared to sodium selenite. No significant differences were observed between all the bacterial Se sources and sodium selenite in SELW1 expression.

Therefore, different Se sources change the mRNA expression of broiler liver selenoproteins, and the effects differ substantially between different selenoproteins, suggesting that some are more sensitive to changes in Se intake than others [51]. This finding is confirmed by the study of Yuan et al. [11], who studied the effect of various Se sources on the expression of liver (GSH-Px1 and TXNDR1) in broiler chicken, and reported that Se yeast and selenomethionin as a sources of organic Se increased GSH-Px1 and TXNDR1 mRNA in the liver compared with sodium selenite. Besides that, there was a considerable increase (*P* < 0.01) in TXNDR1 and SELW1 mRNA level in the group of broiler chickens fed selenomethionin compared to the group that received sodium selenite [52]. The observed difference between organic and inorganic Se may be due to the fact that Se supplied via organic forms has a higher bioavailability and thus enhances the Se level, leading to the stimulation of selenopeoteins gene expression [16]. However, the mechanisms of how different sources of Se can regulate the expression of selenoproteins are still unclear and need more investigation. We note that all Se sources in this study resulted in significant changes in gene expression of liver selenoproteins. However, the bacterial organic Se showed significant differences between the different strains. This may be due to the variation in the type of organic Se compound accumulated in bacterial strains. Organic Se compounds include selenomethionine, selenocystein, and Se-methyl-Secysteine [53], which vary according to their availability to the body.

## Conclusion

To summarize, our study showed that basal diets supplemented with 0.3 mg/kg of different sources of bacterial organic Se and sodium selenite as inorganic source increased the serum and tissues anti-oxidative capacity and Se concentration, up-regulated some selenoproteins mRNA levels, and reduced serum AST, ALT and creatinine level. However, bacterial organic Se showed better effect than sodium selenite in most investigated parameters, and Se extracted from ADS18 bacterial strain had a superior action in improving antioxidant system and expression of selenoproteins compared to ADS1 and ADS2 bacterial Se.

## Abbreviations

A/G: Albumin/ globulin ratio
ADS1: *Enterobacter cloacae*
ADS18: *Stenotrophomonas maltophilia*
ADS2: *Klebsiella pneumonia*
AL: Albumin
ALT: Alanine aminotransferase
AST: Aspartate aminotransferase
BUN: Blood urea nitrogen
BW: Body weight
CAT: Catalase
Cr: Creatinine
DIO1: Iodothyronine deiodinase1
DIO2: Iodothyronine deiodinase2
FI: Feed intake
G: Globulin (G)
GAPDH: Glyceraldehyde-3-phosphate dehydrogenase
GSH-Px: Glutathione peroxidase
GSH-Px1: Glutathione peroxidase1
GSH-Px4: Glutathione peroxidase4
LDH: Lactate dehydrogenase
MDA: Malondialdehyde
Se: Selenium
SELW1: Selenoproteins w
SOD: Superoxide dismutase
T-AOC: Total antioxidant
TP: Total protein
TXNDR1: Thiroxine reductase
WG: Weight gain
